# The multifaceted roles of long noncoding RNAs in pancreatic cancer: an update on what we know

**DOI:** 10.1186/s12935-020-1126-1

**Published:** 2020-02-05

**Authors:** Wenjia Zhou, Lu Chen, Chao Li, Rui Huang, Mian Guo, Shangwei Ning, Jingjing Ji, Xiaorong Guo, Ge Lou, Xinqi Jia, Junjie Zhao, Feng Luo, Chunlong Li, Zhaowei Qu, Shan Yu, Sheng Tai

**Affiliations:** 1 Department of Hepatopancreatobiliary Surgery, The Second Affiliated Hospital of Harbin Medical University, No. 246 XueFu Avenue, Harbin, 150086 People’s Republic of China; 2 Department of Pathology, The Second Affiliated Hospital of Harbin Medical University, No. 246 XueFu Avenue, Harbin, 150086 People’s Republic of China; 3 Department of Orthopedics, The Second Affiliated Hospital of Harbin Medical University, Harbin, China; 4 Department of Colorectal Surgery, The second Affiliated Hospital of Harbin Medical University, Harbin, China; 5 Department of Neurosurgery, The Second Affiliated Hospital of Harbin Medical University, Harbin, China; 6 College of Bioinformatics Science and Technology, Harbin Medical University, Harbin, China; 7 Department of Hepatobiliary and Pancreatic Surgery, Harbin Medical University Cancer Hospital, Harbin, China

**Keywords:** Long noncoding RNAs, Pancreatic cancer, Mechanisms, Biomarkers

## Abstract

Pancreatic cancer (PC) is one of the leading causes of cancer-related deaths worldwide. Due to the shortage of effective biomarkers for predicting survival and diagnosing PC, the underlying mechanism is still intensively investigated but poorly understood. Long noncoding RNAs (lncRNAs) provide biological functional diversity and complexity in protein regulatory networks. Scientific studies have revealed the emerging functions and regulatory roles of lncRNAs in PC behaviors. It is worth noting that some in-depth studies have revealed that lncRNAs are significantly associated with the initiation and progression of PC. As lncRNAs have good properties for both diagnostic and prognostic prediction due to their translation potential, we herein address the current understanding of the multifaceted roles of lncRNAs as regulators in the molecular mechanism of PC. We also discuss the possibility of using lncRNAs as survival biomarkers and their contributions to the development of targeted therapies based on the literature. The present review, based on what we know about current research findings, may help us better understand the roles of lncRNAs in PC.

## Introduction

Pancreatic cancer (PC) is a fatal malignancy with quite stable global morbidity and mortality rates compared with those of other cancers over the past two decades [1, 2]. According to cancer statistics, PC patients have a 5-year survival rate of only 8%, and this dismal prognosis has not improved in years [3]. With the prolonged survival of patients with other major cancers [4], less than 5% of patients with PC are alive after 5 years, even after treatment with surgical resection (up to 20%) [5]. Currently, advanced PC patients still undergo adjuvant therapy consisting of radiotherapy or chemotherapy [2]. The high fatality is mainly attributed to metastasis and easily acquired resistance to these therapies [6]. Therefore, new therapeutic targets and sensitive biomarkers are urgently needed [7]. Although several genes and pathways have been found to participate in the occurrence and progression of PC, the underlying mechanisms are still unclear. Therefore, it is extremely important to investigate novel and effective regulatory models for the further treatment of PC [8].

Long noncoding RNAs (lncRNAs) are defined as noncoding RNAs of more than 200 nt in length and were originally discovered through the large-scale sequencing of mouse cDNA libraries [9–11]. They have attracted intense attention in recent years, as evidence shows that lncRNAs are involved in multiple cancer biology hallmarks [12]. In addition, lncRNAs have been widely reported to participate in the transcriptional, posttranscriptional and epigenetic gene regulation of tumor carcinogenesis. Several studies have also suggested that lncRNAs directly contribute to PC development and progression, as the aberrant expression of lncRNAs has been found in serum or tumor samples; therefore, lncRNAs have great potential as therapeutic targets for PC [10, 13, 14]. In this review, we will address the current understanding of lncRNAs and provide an overview of their potential applications in PC.

## LncRNA classification and function

The discovery of lncRNAs has revealed a new dimension in the pathogenesis of many diseases, including cancer [15, 16]. As players in the ideal RNA regulatory mechanism, lncRNAs are believed to have a great impact on regulating the expression of downstream target genes [17]. Since the abnormal expression and dysfunction of lncRNAs are closely associated with human malignant tumor formation [18, 19], understanding how lncRNAs regulate gene expression at the chromatin remodeling, transcriptional, posttranscriptional and protein levels is becoming extremely important for cancer research [20, 21].

LncRNAs are currently broadly classified into the following categories [20]: (1) antisense RNAs are located within exons and are transcribed from the opposite direction [22]; (2) bidirectional RNAs, similar to antisense RNAs, have a reverse transcription start site but are frequently located within 1 kb of the promoter region of the protein-coding mRNA [23]; (3) long intergenic RNAs (lincRNAs) are independently transcribed noncoding RNAs that do not overlap with annotated protein-coding genes (lincRNAs were identified by studies using tiling arrays of genomic sequences) [24]; and (4) sense intronic RNAs, which are defined as having transcription start sites in introns and ending before exon regions. These RNAs can act as cis-acting agents to regulate adjacent genes on the same chromosome and as trans-acting elements, causing epigenetic changes [14].

Regarding the targeting regulatory mechanisms, lncRNAs possess the following four functions: (1) regulate the spatiotemporal expression of target genes (signal function); (2) act as adapters in functional protein complexes (scaffold function); (3) bind to specific proteins and direct the localization of the resultant complex (guide function); and (4) prevent other RNAs or proteins from binding to their natural targets (bait function). Cis- and trans-acting lncRNAs (such as XIST and HOTAIR, respectively) acting as scaffold molecules can directly recruit target genes for histone- or chromatin-modifying complexes and reduce their expression.

The epigenetic modification of lncRNAs is accomplished through transcription and requires the transcriptional initiation, overlapping expression or RNA-dependent regulation of nearby genes, and a typical example is the case of the lncRNA XIST [25]. Another mechanism of target gene regulation is achieved by binding to chromatin complexes or causing histone epigenetic modifications, which is the case for the lncRNA HOTAIR [4]. LincRNAs may act as enhancers and actively regulate the transcription of proximal genes [26].

LncRNAs direct binding to chromatin remodeling complexes through specific regions functionally as enhancers or mediators of long-range chromatin interactions, thus regulating cis or trans gene transcription [20]. For example, the lncRNA ZEB1 was found to induce metastasis through epithelial-mesenchymal transition (EMT) by inhibiting E-cadherin gene transcription [27, 28]. Moreover, the first lncRNA discovered to be involved in the regulation of transchromatin was HOTAIR [25]. A genome-wide analysis identified hundreds of loci on different chromosomes that interact with HOTAIR [29]. Since they are transcribed from the opposite strand of a protein- or nonprotein-coding gene, such lncRNAs are also termed natural antisense transcripts (NATs) and act as postregulatory factors in gene expression or play a regulatory role through chromatin remodeling [30]. Cis- and trans-acting lncRNAs (such as XIST and HOTAIR, respectively) can function as scaffold molecules to directly recruit histone- or chromatin-modifying complexes to target genes and reduce their expression.

For posttranscriptional regulation, lncRNAs modulate gene expression by the classical “sponge” function, whereby lncRNAs bind to miRNAs and block the binding of the miRNA to its target mRNA, thus promoting the expression of the downstream target mRNA [31]. For example, there is a positive linear correlation between MALAT-1 and LC3B mRNA expression. After MALAT-1 silencing, the major autophagy flux-related genes LC3, LAMP-2 and P62 are significantly altered. Mechanistically, the knockdown of MALAT-1 can release its sponged HuR protein, which in turn leads to the posttranscriptional regulation of T cell intracellular antigen 1 (TIA-1) and has an effect on the inhibition of autophagy [32]. We also summarize current research progress on the “sponge” functions of lncRNAs with miRNAs in Table 1. Moreover, lncRNAs can selectively cleave mRNAs. The most prominent example of this is MALAT-1, an important complex component of the splicing mechanism that binds to and controls the activity of serine/histidine-rich proteins [33]. LATS1 is the main switch in the Hippo-YAP signaling pathway that acts by controlling YAP1. The silencing of MALAT-1 causes the cleavage of LATS1 and affects YAP1. The change in MALAT-1 levels results in the modified proliferation and invasion of AsPC-1 cells upon activation of the Hippo-YAP signaling pathway [34].

**Table 1:** Interacting miRNAs and lncRNAs in pancreatic cancer

LncRNA	Genomic localization	Interacting miRNA	References
HOTAIR	12q12.13	miR-141	[93]
MALAT-1	11q13.1	miR-200c-3p	[19]
H19	11p15.5	miR-675 miR-194	[58, 94]
PVT1	8q24.21	miR-20a-5p	[88]
GAS5	1q25.1	miR-221/SOCS3	[86]
NEAT1	–	miR-506-3p	[64].
SNHG 16	–	miR-218-5p	[61]
ZEB2-AS1	-	miR-204	[79]
TUG1	22q12.2	miR-29c	[73, 74]
XIST	–	miR-141-3p	[72]
MEG3	–	miR-183	[91]
DLEU2	–	miR-455	[12]
HULC	6p24.3	miR-15a	[68]

LncRNAs are also critical for regulating cellular biological processes by binding to RNA-associated proteins [35]. LncRNAs contain multiple functional domains, enabling the sequestration of different intracellular molecules and acting as protein-binding complex adaptors [36]. In addition to sponging miRNAs, lncRNAs can also bind to kinase proteins or DNA through conformational alterations within their domains [37]. Furthermore, lncRNAs can change the alternative splicing of an exon or affect promoter activity. For example, the long noncoding RNA highly upregulated in liver cancer (HULC) can specifically bind to the Y-box binding protein 1 (YB-1) promoter, thus silencing the mRNA of this gene. Additionally, by promoting YB-1 protein phosphorylation, HULC helps release YB-1-bound mRNAs, accelerating tumorigenesis [38].

Recently, it has been found that exosomes use lncRNAs as signal carriers to transmit signals between cells. The release of exosomes into the extracellular environment can be used by tumor cells to change the tumor microenvironment or to affect distant organs for distant metastasis [39]. In addition, many lncRNAs have been found to regulate immune cell differentiation during immune initiation and activation. For example, the lncRNA CECR7 regulates CTLA4 expression in PC by targeting miR-429 [40]. Lnc-DC directly binds to cytoplasmic STAT3 and promotes STAT3 phosphorylation on tyrosine-705, thereby preventing STAT3 from being dephosphorylated by SHP1 [41]. Recent evidence suggests that noncoding RNAs are generally involved in the regulation of host immune responses, especially in the tumor microenvironment [42, 43]. Several lncRNAs play a key role in the regulation of the immune response during tumorigenesis, including those involved in the programmed cell death 1 (PD-1)/programmed cell death ligand 1 (PD-L1) pathway [44]. SNHG20 promotes tumorigenesis through the ATM-JAK-PD-L1 pathway in esophageal squamous cell carcinoma (ESCC) [45]. In conclusion, lncRNAs play an important role in regulating the differentiation and function of immune cells.

## LncRNAs related to PC

**Fig. 1:**
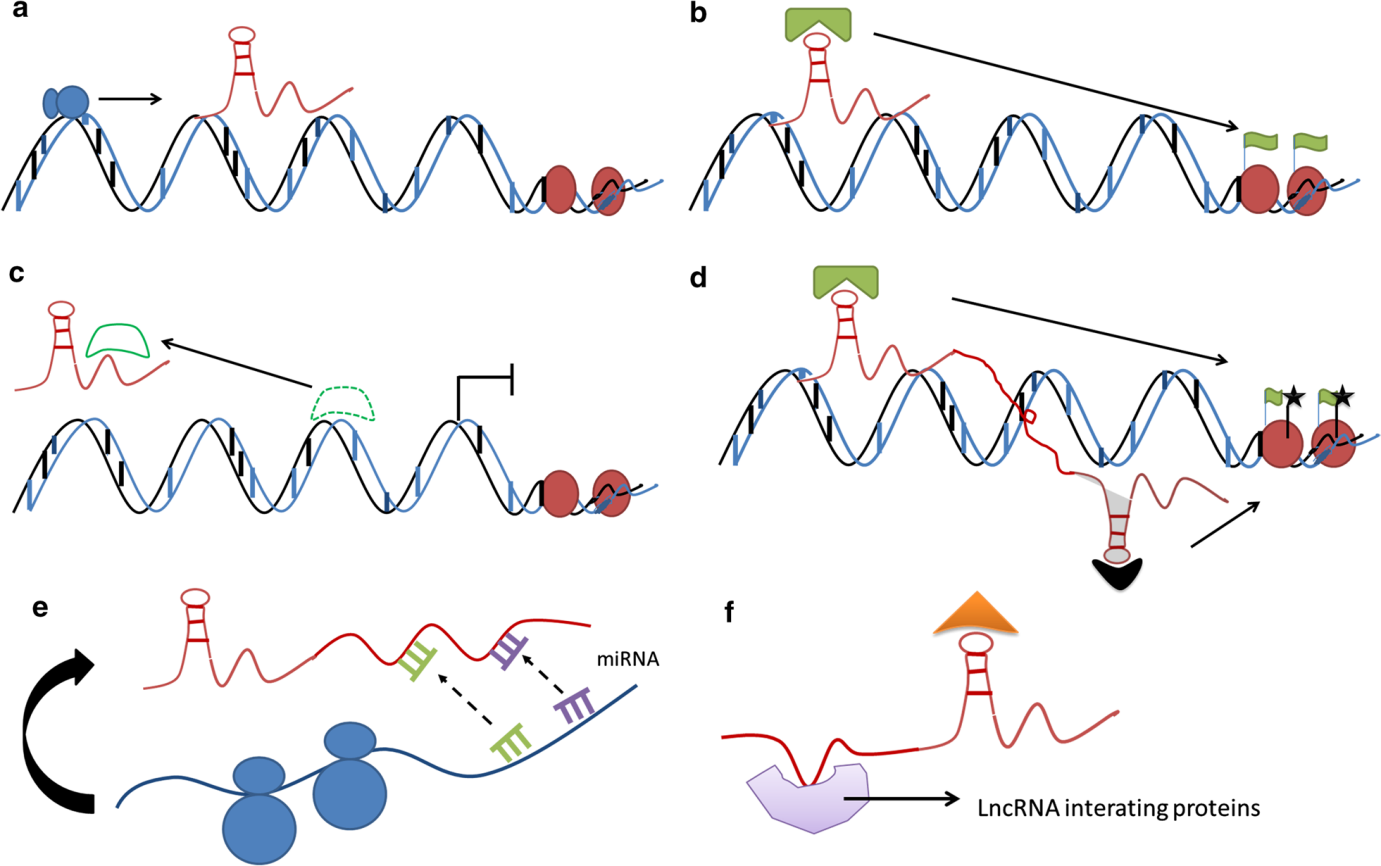
Current understanding of the lncRNA mechanisms involved in PDAC. **a** LncRNAs can function together with transcription factors to regulate target genes. **b** LncRNAs can guide and recruit chromatin-modifying enzymes to target genes. **c** LncRNAs interact with transcription factors or other protein factors to regulate transcription initiation. **d** LncRNAs aggregate a variety of proteins to form a rib nucleoprotein complex that affects histone modifications. **e** LncRNAs sponge functional miRNAs. **f** LncRNAs recruit effector molecules as adaptors to form protein complexes

Pancreatic cancer is also known as the “King of cancers” due to the lack of early diagnostic biomarkers and effective therapeutic methods in advanced stages [46]. LncRNAs have emerged as novel potential therapeutic target tools, and studies have demonstrated that lncRNAs are significantly associated with the progression of PC [47]. For example, high DLEU2 levels indicate a poor prognosis for PC patients [12]. SNHG1 inhibits PC cell proliferation, invasion and metastasis by inhibiting the Notch-1 signaling pathway [48]. Here, we systematically address our understanding of the biological functions of lncRNAs in the PC field (Fig. 1).

### LncRNAs related to PC behavior

#### HOTAIR

HOTAIR is the most well-characterized lncRNA in cancers [49]. HOTAIR is transcribed from the human HOXC locus on chromosome 12 and interacts with the catalytic subunit Ezh2 of PRC2 [50–52]. The major function of HOTAIR in PC is to promote cell proliferation and metastasis [53]. HOTAIR is expressed at low levels in normal pancreatic tissues but is increased in PC tissues [4]. HOTAIR/miR-613/Notch3 is an effective antitumor axis in PC [49]. Further investigation revealed that HOTAIR is responsible for the genome reorientation of polycomb repressive complex 2 (PRC2), partially affecting H3 lysine 27 methylation, which in turn causes PC metastasis [53].

#### HOTTIP

Similar to HOTAIR, HOTTIP has a strong association with the chromatin-modifying complex PRC2-WDR5/MLL1. As an increased level of HOTTIP has been found in PC cells, HOTTIP tends to promote PC stem cell proliferation through WNT/beta-catenin pathway activation by targeting HOXA9 through the complex. Opposite effects, including those on apoptosis and migration, were observed in Panc-1 cells by knocking down HOTTIP [54, 55]. The downregulation of HOTTIP-induced apoptosis was confirmed by enhanced caspase-3/8 activity and Bax family protein expression in PC cells. In addition, mGluR1 levels also decrease with inactivation of the phosphoinositide 3-kinase (PI3K)/AKT/mTOR pathway and may also contribute to PC cell apoptosis [56]. In addition, there is evidence showing that the functional rs1859168 A>C polymorphism may decrease the risk of PC genesis by downregulating HOTTIP expression [57]. Overall, HOTTIP has a considerable impact on PC cell apoptosis.

#### H19

The lncRNA H19 is another WNT signaling activator in PC. H19 and PFTK1 are both upregulated in pancreatic ductal adenocarcinoma (PDAC) samples, and the association between H19 and PFTK1 was discovered along with the functional microRNA-194. Bioinformatics analysis showed that miR-194 has a negative correlation with both PFTK1 and H19. In vitro experiments confirmed the predicted interaction between their nucleotide sequences. The observed inhibition of PDAC proliferation suggested that the H19/miR-194/PFTK1 axis in the WNT signaling pathway contributes to PC proliferation and migration [58]. In vivo experiments showed that the stable knockdown of H19 significantly suppressed the lung and liver metastatic potential of xenografts [8]. These findings imply that H19 plays an important role in proliferation and metastasis in PC.

#### SNHG15 and SNHG16

Small nucleolar RNA host gene 15 (SNHG15) and 16 (SNHG16) are both significantly increased in PC cells [59]. The upregulation of SNHG15 is significantly associated with tumor size, lymph node metastasis, and advanced TNM stage in patients with PC. Functionally, the SNHG15 combination attenuates cell proliferation and simultaneously induces apoptosis and cell cycle arrest at the G0/G1 phase [59]. SNHG15 downregulates P15/KLF2 through EZH2-mediated H3K27me3, contributing to proliferation [60]. Liu and colleagues observed that high SNHG16 levels are associated with poor overall survival [61]. Regarding the mechanism involved, high mobility group box 1 (HMGB1) is a downstream target of miR-218-5p, which can be sponged by SNHG16, causing PC progression [62].

#### Xloc-000647

XLOC-000647 is downregulated in PC and is correlated with tumor stage, the metastasis status and final clinical outcome. A significant negative correlation was observed between the levels of XLOC_000647 and its proximal gene NOD-like receptor family pyrin domain-containing 3 (NLRP3). NLRP3 regulates PC proliferation, invasion and EMT, while XLOC_000647 overexpression reverses the corresponding phenotypes by binding to the NLRP3 promoter [63]. XLOC-000647 is associated with TNM stage and lymph node metastasis and is an independent prognostic factor in patients with PC.

#### DLEU2

DLEU2 levels are indicative of a poor prognosis for PC patients. Real-time PCR results have also revealed that DLEU2 is significantly upregulated in PC samples. A bioinformatics prediction and subsequent luciferase assay confirmed that DLEU2 can bind to miR-455, which leads to cell growth and invasion. As miR-455 is a posttranscriptional regulator of SMAD2, the recovery of SMAD2 from miRNA-mediated inhibition increased PC cell proliferation and invasion, whereas the silencing of DLEU2 attenuated this effect. However, DLEU2 is not an independent biomarker for PC prognosis. Thus, the regulation of DLEU2/miR-455/SMAD2 can be considered a PC therapeutic axis [12].

#### NEAT1

Increased NEAT1 expression is closely related to the survival time of PC patients. NEAT1 mainly participates in cell proliferation by regulating PC apoptosis and arresting the cell cycle. miR-506-3p, a sponge target of NEAT1, inhibits PC cell proliferation but is partially controlled by NEAT1 [60, 64]. These results indicate that the role of carcinogenic lncRNAs may be a new therapeutic target for PC. However, scientists have also found a controversial result, whereby NEAT1, a P53-related lincRNA, shows a suppressive function in PC [65]. NEAT1 deficiency leads to the enhanced transformation of fibroblasts expressing oncogenes and further promotes the initiation of precancerous pancreatic intraepithelial neoplasia (PanIN) in mice overexpressing KrasG12D [65]. Overall, NEAT1 plays an oncogenic or a tumor suppressive role, needing further exploration. However, the loss of NEAT1 may contribute to tumor formation.

#### IRAIN

A study of a small cohort of PC patients reported that IRAIN was closely associated with clinical characteristics, including size, the TNM status and metastasis [66]. The silencing of IRAIN increases the apoptosis of BxPC-3 and Panc-1 cells. Regarding the underlying mechanism involved, IRAIN has strong binding affinity to the histone demethylase lysine-specific demethylase 1 (LSD1) and downregulates Kruppel-like factor 2 (KLF2) and P15, which in turn results in PC cell death by causing significant apoptosis.

#### HULC

HULC was first identified as a carcinogenic lncRNA in liver cancer [67]. In addition to its functions in hepatic cancer, HULC overexpression also promotes PC growth and invasion. However, a rescue experiment showed the opposite phenotype, with a significant increase in apoptosis. Similar to other lncRNAs, HULC can downregulate miR-15a in the Panc-1 cell line by activating the PI3K/AKT pathway [68].

#### ENST00000480739

ENST00000480739, a novel lncRNA, inhibits PDAC cell invasion. A luciferase assay confirmed that ENST00000480739 can bind to the promoter of OS-9, increasing OS-9 mRNA levels and targeting oxygen-inducible factor-1a (HIF-1a). These findings were validated in 35 PDAC patients, in which ENST00000480739 was frequently downregulated [69].

#### DUXAP8

The expression of the pseudogene-derived lncRNA DUXAP8 is higher in PC tissues than in adjacent normal pancreatic tissues. It has also been reported that DUXAP8 can be used as an independent biomarker to predict pathology, the TNM stage and clinical outcomes [70]. CDKN1A and KLF2 have been confirmed as downstream targets of DUXAP8. However, the interaction between DUXAP8 and CDKN1A/KLF2 only partially contributes to PC proliferation. The same study found that DUXAP8 can epigenetically silence CDKN1A and KLF2 mRNAs by combining with EZH2 or LSD1. However, further research may be needed to investigate the direct target genes of DUXAP8 and the possible mechanisms involved.

#### XIST

XIST overexpression significantly promotes PC tumor growth, invasion and migration. miR-34a-5p is a target gene of XIST and is associated with poor survival [25]. XIST-mediated cancer-aggressive behaviors can be strongly attenuated by miR-34a-5p mimics both in vivo and in vitro [71]. These findings indicate that the XIST/miR-34a-5p axis has antitumor potential in PC. In addition, XIST can direct miR-141-3p binding and mutual inhibition. miR-141-3p can also negatively regulate TGF-2 in PNCA-1 cells. XIST overexpression attenuates the inhibitory effect of miR-141-3p on TGF-2 and promotes the proliferation, migration and invasion of PC cells via the miR-141-5P/TGF-2 axis [72].

#### PCTST

PCTST expression is lower in PC samples than in normal control samples. Increasing PCTST expression affects the EMT process through the upregulation of E-cadherin and the suppression of vimentin. In addition, silencing TACC-3 can mimic the effect of PCTST upregulation. It is worth noting that PCTST is significantly related to its proximal gene TACC-3 and binds to the promoter of TACC-3 to suppress TACC-3 expression. Therefore, the lncRNA PCTST acts as a tumor suppressor gene that regulates tumorigenesis and EMT by targeting the promoter of TACC-3 in PC [73].

#### TUG1

Taurine upregulated 1 (TUG1) is upregulated in PC samples, and TUG1 knockdown exerts a significant tumor suppressor effect on PC behaviors [74]. Further investigations revealed that TUG1 can directly target miR-29c, a well-known tumor suppressor miRNA in cancers. TUG1 downregulation increases miR-29c functions. Moreover, downstream effectors of miR-29c, including integrin subunits β1, MMP2 and MMP9, are significantly decreased. The upregulation of miR-29c reverses the TUG1 knockdown-mediated inhibition of tumor growth [73]. Overall, via its effect on miR-29c, the inhibition of TUG1 may represent an effective therapeutic method for PC.

#### PVT1

Xie et al. reported that the PVT1 expression level may be used to detect the ductal PC status and may have better sensitivity and specificity than serum CA199 [75]. Wu et al. noted that PVT1 expression was dramatically upregulated in PC tissues and cell lines [76]. PVT1 acts as a “molecular sponge” to inhibit the activation of miR-448 and promote the proliferation and metastasis of PC cells, thereby inhibiting the effect of SERPINE1 mRNA-binding protein 1 on target cells [77]. In addition, the authors found that PVT1 stimulated cell proliferation and EMT by downregulating p21 in PC cells. Because p21 can mediate the expression of ZEB1 and Snail in EMT-related pathways, further study is needed to confirm the regulatory network to prove the therapeutic effect of PVT1 on PC [78].

#### ZEB2-AS1

ZEB2-AS1 has been explored only as a competing endogenous RNA (ceRNA) and was shown to sponge miR-204, further reducing the proliferation and invasion of PC cells. According to the report, HMGB1 is the main downstream target of miR-204. The evaluation of the ZEB2-AS1/miR-204/HMGB1 axis for PC treatment is still in its infancy [79].

### Diagnostic, therapeutic and prognostic implications of LncRNAs in PC

Sensitive and effective biomarkers for PC remain largely unexplored, and we collected up-to-date research findings on lncRNAs in PC (Table 2). Although intensive efforts have been made at the mRNA and protein levels, PC is still lacking in this area. As the understanding of noncoding RNAs has improved, several lncRNAs related to PC have been found to have the potential to be possible effective markers [80].

**Table 2:** LncRNAs as biomarkers of pancreatic cancer

Biomarkers	Early diagnosis	Prognostic value	Sample resources
LncRNA	HOTAIR↑	HOTAIR↑	Tumor
	MALAT-1↑	MALAT-1↑	Tumor
	HOTTIP↑	HOTTIP↑	Tumor
	PVT1↑	PVT1↑	Saliva
		H19↑	Tumor
		GAS5↓	Plasma
		ENST00000480739↓	Tumor
		BC008363↓	Tumor

The arrows (up or down) in the table indicate the increase or decrease in lncRNA expression and a poor prognosis or a malignant diagnosis

#### HOTAIR

Jiang et al. demonstrated that silencing HOTAIR affected autophagy by increasing ATG7 and WIF-1 [81]. Because ATG7 and WIF-1 are both major genes involved in the WNT pathway, which regulates radiosensitivity, the HOTAIR/ATG7/WIF-1 axis can be considered a novel therapeutic target for enhancing PDAC radiation therapy [82]. In addition, another research report by Yu et al. showed that the expression levels of HOTAIR and hexokinase-2 (HK2) were both increased in tumor tissues and serum. The serum levels of these two factors can be accurately used for the early diagnosis and prognosis of PC [83].

#### ENST00000480739

In a small cohort study of 35 PDAC patients, decreased ENST00000480739 expression, with a reduction of at least 50%, was found in 66% of PDAC tissues, and ENST00000480739 showed a negative correlation with lymph node metastasis. ENST00000480739 was also identified as an independent prognostic marker of the overall survival time in patients who underwent surgeries [69].

#### BC008363

The lncRNA BC008363 is detected at extremely low levels (approximately 23-fold lower) in ductal pancreatic invasive carcinoma samples, which strongly suggests that its expression level can be used as a distinguishing marker for invasive lesions or carcinomas in situ. Experimental results have shown that patients with high BC008363 expression have a much longer survival time than patients with low BC008363 expression, indicating its independent prognostic value in PDAC [84]. Therefore, further studies on BC008363 are required to validate its prognostic and diagnostic potential for PC.

#### GAS5

The lncRNA growth arrest-specific 5 (GAS5) plays a significant tumor suppressor role not only in PC but also in various other cancers [85]. Evidence of the involvement of GAS5 in the chemoresistance of PC cells has also been found, whereby GAS5 can sponge miR-181c-5p and inhibit Hippo signaling. GAS5 overexpression facilitates the chemotherapeutic effect in a mouse model of PC [86]. Other reports have shown that GAS5 contributes to drug-induced apoptosis through PTEN regulation by miR-32-5p in different PC cell lines [87]. Jennifer et al. successfully quantified 28 lncRNA mutants in preoperative archived plasma from a cohort of pathologically confirmed intraductal papillary mucinous neoplasm (IPMN) cases and unaffected controls [26]. Their results showed that GAS5 could differentiate IPMN samples from healthy samples. This finding provides novel information on the ability to detect intracellular RNA from IPMN patients and suggests that GAS5 may serve as a secondary diagnostic marker for the identification and pathological classification of IPMN.

#### Malat-1

Metastasis-associated lung adenocarcinoma transcript-1 (MALAT-1) is a lncRNA that is an independent negative prognostic marker for PC patients [32]. Similar to its name, MALAT-1 has been shown to be associated with a poor prognosis in PC. Kaplan–Meier analysis showed that the overall survival time of patients with PDAC expressing high levels of MALAT-1 was significantly lower than that of patients expressing low levels of MALAT-1. A multivariate analysis supported the notion that the MALAT-1 level is an independent prognostic factor for PDAC [19].

#### Pvt1

PVT1, as a ceRNA, competes with endogenous miR-20a-5p to increase the expression of ULK1 protein, which is upregulated in a variety of tumor types and associated with tumor progression and chemoresistance [88]. These results reveal the potential role of PVT1 in autophagy regulation and PDAC cell growth and suggest that the “PVT1/miR-20a-5p/ULK1/autophagy” axis is a novel therapeutic target for PDAC.

## Conclusion and future perspectives

**Fig. 2:**
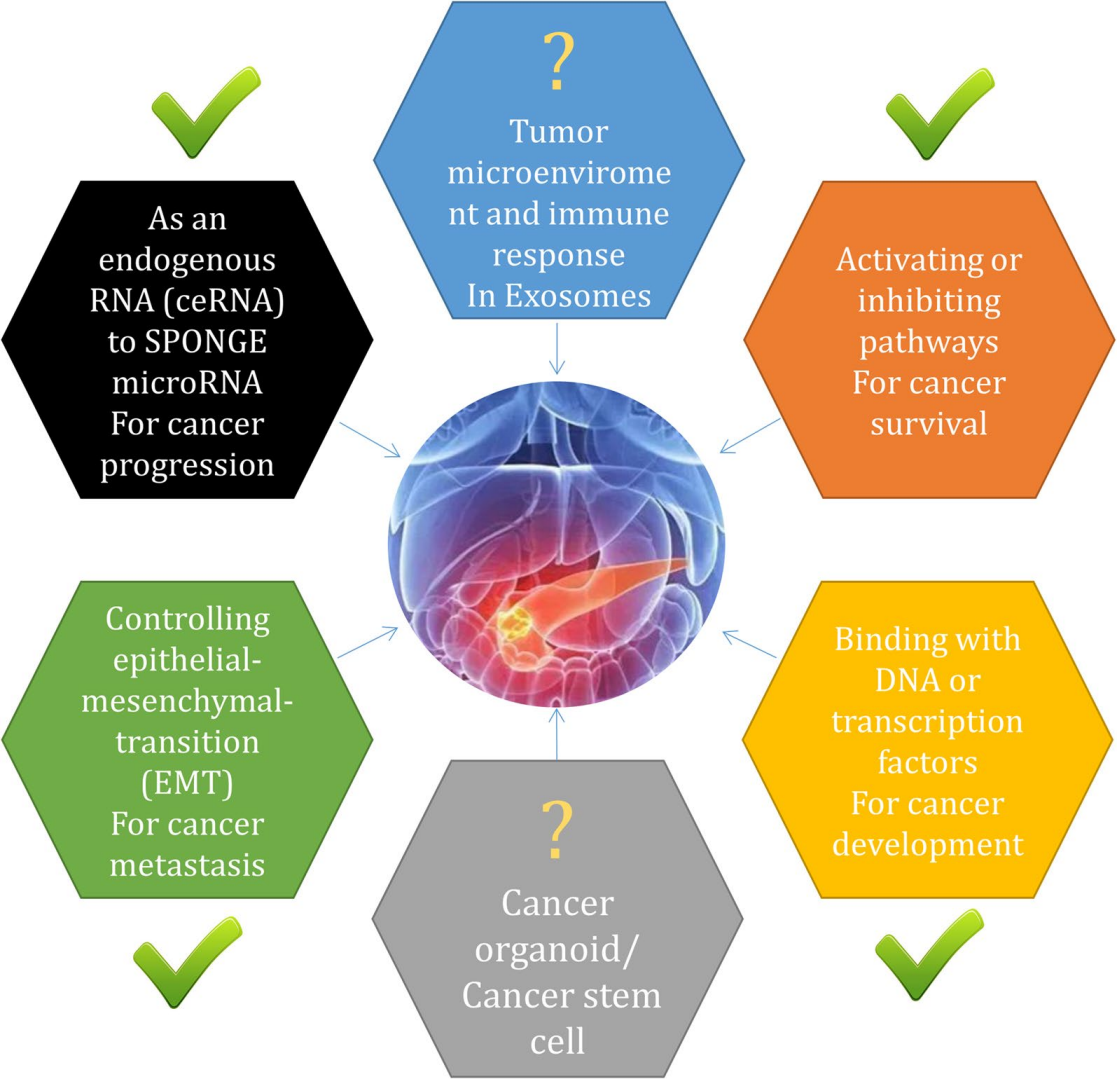
Current research progress on the biological functions of lncRNAs in pancreatic cancer and future investigation directions

LncRNAs were originally thought to be the “noise background” of transcription. As a deeper understanding of transcripts improved our knowledge of RNAs, an increasing number of cancer-related experiments have demonstrated that lncRNAs are important regulators with different mechanisms in PC (Fig. 2). However, there are still contradictory results for specific RNAs, which may be due to inconsistent models or different downstream targets and specific mechanisms to maintain the same phenotypes (listed in Table 3). In addition, the current study designs are mostly relatively simple, while comprehensive networks are much more difficult to fully investigate. Although significant effects have been observed at the cellular and organismal levels, many important issues regarding the complexity of lncRNAs remain to be resolved. Given their biological versatility, it is important to know whether lncRNAs are activated or silenced in any given tissue and how they perform their respective functions. Although we have begun to understand some of the effects of lncRNAs, new features remain unexplored, such as the positive and negative feedback regulatory mechanisms of lncRNA expression. Our current level of knowledge is still greatly limited by methodology, and additional progress is needed to develop appropriate approaches to enable researchers to study the structure and characterize the spatial and developmental specificity of lncRNAs. Other obstacles are based on the in vivo suppression or activation of lncRNAs. The lack of specific organ targets, susceptibility to degradation in cells and low efficiency of delivery are obstacles that still need to be overcome.

**Table 3:** LncRNAs dysregulated in pancreatic cancer

LncRNA	RNA description	Expression in PC	Functions in cancers	References
HOTAIR	LncRNA HOX transcript antisense RNA	Up	Related to cancer cell proliferation, progression and invasion	[4]
HOTTIP	HOXA transcript at the distal tip	Up	Promotes cancer cell proliferation, inhibits cell apoptosis, increases migration	[54]
MALAT-1	Metastasis-associated lung adenocarcinoma transcript 1	Up	Regulates the cell cycle, growth, migration and invasion	[19]
H19	LncRNA H19	Up	Enhances pancreatic cancer cell proliferation	[90]
ENST00000480739	LncRNA ENST00000480739	Down	Cell proliferation, invasion and migration	[69]
BC008363	LncRNA BC008363	Down	Potential critical role in tumorigenesis	[84]
PVT1	Plasmacytoma variant translocation 1	Up	Unknown	[75]
GAS5	Growth arrest-specific 5	Down	Inhibits cell proliferation	[86]
NEAT1	p53-inducible lincRNA	Down	Promotes neoplasia	[65]
SNHG 15 16	Small nucleolar RNA host gene	Up	Regulation of cell proliferation, migration and invasion of tumors	[59, 61]
ZEB2-AS1	LncRNA ZEB2-AS1	Up	Cell growth and invasion	[79]
TUG1	Taurine upregulated 1	Up	Cell proliferation, invasion, and migration	[73, 74]
XIST	LncRNA X inactive-specific transcript	Up	Promotes proliferation, migration and invasion	[25]
MEG3	LncRNA MEG3	Down	Tumor suppressor	[91, 92]
DUXAP8	A pseudogene-derived lncRNA	Up	Cell proliferation and promotes apoptosis in vitro and in vivo	[70]
XLOC-000647	LncRNA XLOC-000647	Down	Cell proliferation and invasion	[63]
IRAIN	LncRNA IRAIN	Up	Cell apoptosis and induces growth arrest	[66]
DLEU2	LncRNA DLEU2	Up	Cell proliferation and invasion	[12]
HULC	LncRNA HULC	Up	Cell proliferation, migration and invasion	[68]
PCTST	LncRNA PCTST	Down	Inhibits cell proliferation, invasion, tumorigenesis and EMT	[73]

LncRNAs are easily detected in body fluids and are widely found in the blood, urine, saliva and even pancreatic fluid. At present, it has been reported that lncRNAs can be found in exosomes from tumors, providing a very promising research direction for the diagnosis and treatment of PC. Moreover, some lncRNAs increase the growth of intestinal epithelial cells and organoids in mice [89]. This experiment highlights the importance of lncRNAs in organoid and cancer cell survival and the potential of lncRNAs as therapeutic targets in a broad spectrum of cancers. In addition to cancer organoids, cancer stem cells and the microenvironment are also important research areas requiring investigation. However, these research directions have not been intensively studied in PC.

In summary, the potential of lncRNAs in the diagnosis and treatment of PC is unquestionable but requires large validation cohorts. Based on the currently known information about lncRNAs in PC, it is still difficult to obtain a clear answer to conclude the functional mechanism of lncRNAs. However, lncRNAs widely participate in regulating cell division, directly causing tumorigenesis, inducing tumor metastasis and actively participating in clinical treatment. The understanding of lncRNA biology is still in its infancy, and the determination of lncRNAs as critical molecules for the development and treatment of PC still has a long way to go. However, the future of the lncRNA field for cancer therapy is undeniably promising.

## Data Availability

Not applicable.

## Abbreviations

EMT: Epithelial-mesenchymal transition
EZH2: Enhancer of zeste homolog 2
GAS5: Growth arrest-specific 5
HOTAIR: HOX transcript antisense RNA
HULC: Highly upregulated in liver cancer
HIF-1a: Hypoxia-inducible factor-1α
HMGB1: High mobility group box 1
HPDE: Human normal pancreatic ductal epithelial
HOXA9: Homeobox A cluster 9
HK2: Hexokinase-2
ITGB1: Integrin subunit β1
IPMN: Intraductal papillary mucinous neoplasm
KLF2: Kruppel-like factor 2
LncRNAs: Long noncoding RNAs
LincRNAs: Long intergenic RNAs
LSD1: Lysine-specific demethylase 1
MALAT-1: Metastasis-associated lung adenocarcinoma transcript1
MLL1: Mixed lineage leukemia 1
MMP2: Matrix metalloproteinase-2
MMP9: Matrix metalloproteinase-9
NATs: Natural antisense transcripts
NLRP3: NOD-like receptor family pyrin domain-containing3
PC: Pancreatic cancer
PCR2: Polycomb repressive complex 2
PDAC: Pancreatic ductal adenocarcinoma
PanIN: Pancreatic intraepithelial neoplasia
PI3K: Phosphoinositide 3-kinase
SNHG: Small nucleolar RNA host gene
SiRNA: Small interfering RNA
SnoRNA: Small nucleolar RNA
TUG1: Taurine upregulated 1
TIA-1: T cell intracellular antigen 1
WDR5: WD repeat containing protein 5
